# Mechanisms of Action of Prebiotics and Their Effects on Gastro-Intestinal Disorders in Adults

**DOI:** 10.3390/nu12041037

**Published:** 2020-04-09

**Authors:** Michele Pier Luca Guarino, Annamaria Altomare, Sara Emerenziani, Claudia Di Rosa, Mentore Ribolsi, Paola Balestrieri, Paola Iovino, Giulia Rocchi, Michele Cicala

**Affiliations:** 1 Gastroenterology Unit, Università Campus Bio-Medico di Roma, via Álvaro del Portillo 21, 00128 Rome, Italy; m.guarino@unicampus.it (M.P.L.G.); s.emerenziani@unicampus.it (S.E.); m.ribolsi@unicampus.it (M.R.); p.balestrieri@unicampus.it (P.B.); giuliarocchi4@gmail.com (G.R.); m.cicala@unicampus.it (M.C.); 2 Unit of Food Science and Human Nutrition, Campus Bio-Medico University of Rome, Via Álvaro del Portillo 21, 00128 Rome, Italy; c.dirosa@unicampus.it; 3 Gastrointestinal Unit, Department of Medicine, Surgery and Dentistry Scuola Medica Salernitana, Università di Salerno, Via Allende, 84081 Salerno, Italy; piovino@unisa.it

**Keywords:** prebiotics, gastro-intestinal disorders, intestinal microbiota, oxidative stress

## Abstract

In recent years, research has focused on the use of dietary fibers and prebiotics, since many of these polysaccharides can be metabolized by intestinal microbiota, leading to the production of short-chain fatty acids. The metabolites of prebiotic fermentation also show anti-inflammatory and immunomodulatory capabilities, suggesting an interesting role in the treatment of several pathological conditions. Galacto-oligosaccharide and short- and long-chain fructans (Fructo-oligosaccharides and inulin) are the most studied prebiotics, even if other dietary compounds seem to show the same features. There is an increasing interest in dietary strategies to modulate microbiota. The aim of this review is to explore the mechanisms of action of prebiotics and their effects on the principal gastro-intestinal disorders in adults, with a special focus on Galacto-oligosaccharides, Fructo-oligosaccharides, lactulose and new emerging substances which currently have evidence of prebiotics effects, such as xilooligosaccharides, soybean oligosaccharides, isomaltooligosaccharides, lactobionic acid, resistant starch and polyphenols.

## 1. Prebiotics

Gibson and Roberfroid elaborated the first definition of prebiotics in 1995 as “selectively fermented ingredients that allow for specific changes, both in the composition of and/or activity in the gastrointestinal microflora that confer benefits upon hosts’ well-being and health” [1]. Afterwards, in 2007, the FAO Technical Meeting on Prebiotics defined the term “prebiotic” as “a non-viable food component that confers a health benefit on the host associated with the modulation of microflora” [2], and that definition has also been integrated into the *Guidelines on Probiotics and Prebiotics* of the Italian Ministry of Health in 2018 [3]. This category of products encompasses primarily short and long chain fructans (Fructo-oligosaccharides (FOS) and inulin), Galacto-oligosaccharides (GOS), and lactulose [1,2,3,4,5]. These compounds, if inserted into a diet in small quantities (5–20 g/day), stimulate the growth of bifidobacteria and lactobacilli, which are not the most abundant microorganisms in the intestine, except in breastfeeding babies [6]. 

The increasing knowledge about prebiotics has increased the number of scientific studies, as well as industrial interest, and this phenomenon has meant that prebiotic function has been associated with a lot of oligosaccharides and polysaccharides, without considering the right criteria [7]. 

Indeed, in 2017, during an important meeting of the International Scientific Association of Probiotics and Prebiotics (ISAPP) [8], prebiotics were identified as “a substrate that is selectively utilized by host microorganisms, conferring a health benefit”. Moreover, these substances have to show specific features, which are to be tested by in vitro and in vivo experiments in different targets (i.e., animals or humans): (1) resistance to gastric acidity, hydrolysis by digestive enzymes and gastro-intestinal (GI) absorption; (2) fermentation by intestinal microflora, which can be evaluated in vitro through the addition of the respective carbohydrates to colon content suspensions, or pure or mixed bacteria cultures in an anaerobic batch or continuous culture fermentation system [9]; and (3) growth promotion of intestinal bacteria beneficially related to health and well-being [10]. This definition appears to be the most complete and currently used definition.

Moreover, according to the FAO, and consequently to the Italian Ministry of Health, prebiotics have also to satisfy these requirements: (1) they have to be safe for both men and women, based on traditional uses, so they cannot be recognized as novel food, pursuant to the regulation (UE) 2015/2283; and (2) they have to be ingested in a plausible daily amount in order to have a “prebiotic” effect, according to scientific evidence [2,3]. 

A normal diet usually contains many prebiotic carbohydrates. For example, inulin-type fructans are present in large amounts in chicory root, Jerusalem artichoke and garlic, but they are also found, in smaller amounts, in cereals, such as wheat [11]. Other carbohydrates, such as soybean oligosaccharides [5], isomaltooligosaccharides, xilooligosaccharides [5], arabinooligosaccharides, lactosucrose, lactobionic acid, resistant starch [12], psyllium and galactomannan, could be present in a complete diet, and it is demonstrated that they also have prebiotic effects [13]. Moreover, according to the ISAPP consensus statement [8], other substances, such as polyphenols and polyunsaturated fatty acids, which are converted to respective conjugated fatty acids, could be included in the most recent definition, acquiring a convincing weight of evidence in the target host [14,15]. 

## 2. Prebiotics and Dietary Fibers

All prebiotics are fibers, whereas not all the dietary fibers have prebiotic effects. The term “dietary fiber” was coined in 1953, but already years before, some properties, such as laxative effects, increasing stool weight and preventing diseases, have been associated with fibers [13]. Nowadays, the association between dietary fibers and the prevention of cardiovascular and metabolic diseases, such as diabetes, obesity and cancer, is well known [13]. 

To date, there is no common definition of dietary fibers. Actually, there are different definitions in the world [13]. According to the Institute of Medicine (IOM) definition of 2001, then adopted by the U.S. Food and Drug Administration and American Dietetic Association (actually, the Academy of Nutrition and Dietetics) in 2008, fibers could be divided into: (1) Dietary fibers that consist of nondigestible carbohydrates and lignin, which are intrinsic and intact in plants; (2) Functional fibers, which are isolated, non-digestible carbohydrates, with beneficial effects on humans [16,17].

In 2009, the Codex Alimentarius Commission gave a dietary fiber definition, and the year after, the Ninth Vahouny Fiber Symposium [18] took place to add some issues to the aforementioned definition. They agreed that undigestible carbohydrates with a degree of polymerization (DP) 3-9 also have to be included among dietary fibers, and they listed the beneficial effects of fiber on human health: (1) A reduction in blood lipid levels; (2) A reduction in post prandial blood glucose and insulin level; (3) An increased stool mass and reduced intestinal transit; (4) Fermentability by microbiota [18].

Finally, a more recent document of the Codex Alimentarius Commission (last amended in 2017) gave another definition of dietary fibers: “they are carbohydrate polymers with ten or more monomeric units, which are not hydrolyzed by the endogenous enzymes in the small intestine of humans and belong to the following categories: edible carbohydrate polymers naturally occurring in consumed food; Carbohydrate polymers, which have been obtained from food raw material by physical, enzymatic or chemical means and which have been shown to have a beneficial physiological effect on health, as demonstrated by the generally accepted scientific evidence of competent authorities; Synthetic carbohydrate polymers, which have been shown to have a beneficial physiological effect on health, as demonstrated by the generally accepted scientific evidence of competent authorities” [19]. 

Traditionally, dietary fibers are divided into categories of soluble and insoluble. The soluble fibers are thought to have beneficial effects on serum lipids, while the insoluble fibers increase stool weight and have laxative effects. 

However, that division is not always supported by scientific evidence. Moreover, fibers are also classified according to viscosity and fermentability. Viscous fibers are able to form a gel in the intestinal tract, while fermentable fibers are metabolized by microbiota. It is important to underline that there is not a strict classification of different kinds of fibers [13].

To better explore the difference between dietary fibers and prebiotics, it is important to remark that human enzymes are not able to degrade several glyosidic linkages present in a subset of polysaccharides, such as cellulose, hemicelluloses, mucilage, pectin and lignin, and those not digested by human enzymes are often partly fermented in the gastrointestinal tract [20]. Some dietary fibers are also able to selectively stimulate the growth and/or activity of intestinal bacteria potentially associated with health and well-being, acting as prebiotics [10].

The ability of dietary fibers consumption in modulating microbiota has been extensively demonstrated. Moreover, it is well know that, in humans, switching between a diet rich in fibers (>30 g/day) to a meat-based diet leads to a fast (within 24 h) shift in bacterial diversity and the production of fermentative end products, even if this is not sufficient to cause a prebiotic effect [21]. At the same time, dietary strategies, aimed to enhance the adherence to the Mediterranean diet, can be associated with health benefits. A regular consumption of fibers is conducive to the maintenance of the beneficial effects; indeed, in the study by Hiel et al. [22], three weeks after the end of inulin-rich vegetables diet, the levels of Bifidobacterium genus and Clostridiales were shown to have returned to the previous levels.

## 3. Mechanisms of Action of Prebiotics

In recent decades, several studies have underlined the health benefits of prebiotics, including effects on the gastrointestinal (GI) tract (i.e., the prevention of pathogen damage or immune system modulation, the improvement of gut barrier function, a reduction in the pathogenic bacteria population, the production of short-chain fatty acids, SCFAs) [13], on the cardiovascular system (i.e., a reduction in blood lipid levels or effects on insulin resistance), on mental health (i.e., metabolites that influence brain function, energy and cognition) and on bone (i.e., mineral bioavailability), etc. [8]. 

Regarding the potential effects on the GI tract, non-digestible carbohydrates, such as prebiotics, can deeply modulate the composition and activity of intestinal microbiota [7,23,24]. 

Generally, in the human intestine, the enzymes that hydrolyze the polymer bonds of these prebiotics are lacking, so they can resist small-intestinal digestion and reach the colon intact, where they undergo fermentation by beneficial bacteria, such as Lactobacilli and Bifidobacteria [25].

As gut microbiota have been involved in the pathogenesis of several GI disorders [20], there is an increasing interest in dietary strategies to modulate microbiota. For this reason, research has focused on the use of prebiotics, since many of these polysaccharides can be metabolized by intestinal microbiota, leading to the production of SCFAs (including acetate, butyrate, and propionate) [26]. The action of prebiotics on microbial diversity in the colon is still under debate. Indeed, the few human studies that have been conducted provided contradictory observations regarding SCFA levels. An interesting study by Liu et al. [27], carried out using healthy volunteers, found a significant decrease in butyrate producers’ levels and an increase in Bifidobacterium levels, after the administration of FOS and GOS, which can be caused by high levels of lactic acid, responsible for creating an environment inhospitable for butyrate producers. A limit of this study is that this intervention was conducted for a period of 14 days, and longer intervention studies may be fundamental to better exploring the effects of prebiotic administration. 

Actually, a specific advantage of prebiotics is the growth of target microorganisms that, in turn, compete with species that are injurious to energy sources and exclude them by protecting or promoting the production of beneficial fermentation substances, such as SCFAs, which have immunomodulatory properties, influencing toll-like receptor-4 signaling and the production of pro-inflammatory cytokines [25]. Among many potential prebiotics assessed, only a few substrates, i.e., inulin, FOS and GOS, have been validated by means of human studies. Nevertheless, fructans are known to be the main substrate of healthy microbes, GOS and lactulose, which seems to determine a major growth of Lactobacilli and Bifidobacteria, compared to inulin [28]. 

Kanner et al. [29] have shown that gastric acid secretion is able to promote the oxidation of lipids and other food substances. According to their studies, dietary antioxidants (including inulin) may play a role in preventing lipid peroxidation in the stomach. In general, dietary supplementation of inulin or oligofructose contributes to protection from oxidative stress, consequently preventing inflammatory reactions associated with oxidative stress [30,31]. 

Moreover, it is interesting to note that the process called “cross-feeding”, in which the metabolism of dietary fibers, mostly prebiotics, from some microbes indirectly stimulates the growth of other ones, and the products of fermentation become, in turn, a substrate for the growth of other bacteria. For example, the products of fermentation of Bifidobacteria and Lactobacilli, the main utilizers of fructans, are lactate and acetate, which can be used as an energy source from other bacteria, including Eubacterium, Roseburia, and Faecalibacterium, that, in turn, produce butyrate. Thus, it has been shown that the dietary consumption of fructans is related to an increase in butyrate levels, even though the primary increases in bacteria, following the consumption of fructans, do not directly produce butyrate [26]. 

Briefly, the mechanisms of action known to date of fructans, GOS and Lactulose are reported in this section, with a final focus on new interesting molecules, which are showing prebiotic effects.

### 3.1. Fructans

The first important action of fructans ingestion on the GI system is the modulation of intestinal microflora: actually several studies have demonstrated that inulin exerts a favorable effect on the level of *F. Prausnitzii* and *Anaerostipes sp.* inside the intestine, which may explain some of the butyrogenic effects resulting from it, when inulin is ingested [32,33,34]. Similarly, FOS and GOS have also been demonstrated to improve *F. Prausnitzii* levels [35]. Interestingly, in a dose–response trial of FOS supplementation in healthy subjects, the dose of 10 g/daily is the minimum dose of prebiotics able to induce a bifidogenic effect [36], while for inulin-type prebiotics the dose is lower (2.5–5 g/day) [37,38].

In fact it has been shown that two weeks of consuming vegetables rich in inulin-type fructans led to a 3.8-fold increase in the Bifidobacterium genus, and, at the species level, it induced an increase in *B. longum subsp. longum* and, to a lesser extent, *B. pseudocatenulatum*, *B. bifidum* and *B. adolescentis* [22]. These results confirm previous data, which showed that the consumption of Jerusalem artichoke (rich in inulin) was related to an increase in Bifidobacterium [39,40]. 

**Figure 1:**
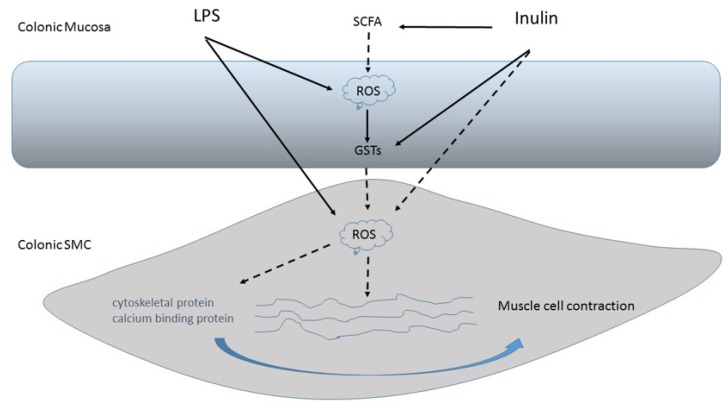
The antioxidant action of inulin-type fructans on colon mucosa and contractility. Inulin, through short-chain fatty acids, can act as a scavenger of reactive oxygen species (ROS). Inulin is also able to modulate responses to pathogenic bacterial insults (Lipopolysaccharide (LPS)) and protect gut from inflammatory processes, probably stimulating defenses against ROS by up-regulating colonic mucosal detoxification enzymes (GSTs), and in this way, inulin restores the level of some important proteins involved in intestinal smooth muscle contraction. Dotted line: inhibitory action. Solid line: stimulatory action. LPS, Lipopolysaccharide. ROS, reactive oxygen species. GSTs, glutathione S-transferases. SCFA, short-chain fatty acids.

Moreover, as recently demonstrated, fructans are also able to show an antioxidant capability higher than sucrose, glucose and fructose [2], suggesting that the antioxidant feature is typical of FOS. Indeed, the fructan antioxidant capability seems to be influenced by the DP and/or the presence of branches in the molecule. In particular, linear fructans with a low DP (i.e., inulin Frutafit IQ^®^) and branched fructans (agavins) seem to have the highest antioxidant capability [38]. Furthermore, the antioxidant capability of inulin IQ appears to be resistant to cooking and digestion. Interestingly, this evidence makes the more intriguing antioxidant feature of fructans, which are generally the main water-soluble antioxidants, quite unstable. It is believed that inulin-type fructans can act indirectly as a scavenger of reactive oxygen species (ROS), thanks to the action of SCFAs, resulting from their fermentation in the colon. Moreover, they can also stimulate the activity of antioxidant enzymes glutathione S-transferases (GSTs) [41]. Therefore, inulin-type fructans could directly act as a powerful scavenger of ROS, blocking the growth and development of pathogens that can be stimulated by the ROS derived from gastro-intestinal anti-inflammatory responses [42,43,44]. We have previously demonstrated the protective effect of inulin on Lipopolysaccharide (LPS)-induced damage of colonic smooth muscle in an ex vivo experimental model, which seems to be related to the presence of oxidative stress [42]. The beneficial effect of inulin on LPS-induced muscle cell impairment is due to the ability of this fructan to contrast the oxidative stress induced by LPS in the human colonic mucosa. Indeed, it has been demonstrated, in a previous study, that the level of protein oxidation induced by LPS exposure was significantly reduced by inulin treatment [42]. In addition, in this study, it was interesting to observe that the antioxidant capability was significantly higher when the colonic mucosa was exposed to LPS and Inu ^®^ IQ, when compared to the LPS exposure alone. This finding strengthens the view that inulin inhibits the release of free radicals (H2O2), and it seems to protect the human colon mucosa from damage induced by LPS (Figure 1).

However, the specific mechanisms by which inulin acts on intestinal muscle function and the molecular mechanisms involved in the direct and/or indirect response of colonic mucosa to this prebiotic are not well understood.

Data from a recent study confirm the protective effect of inulin on LPS-induced colonic mucosal oxidative stress and muscle impairment. Using iTRAQ analysis, it was demonstrated that inulin restored the level of some important protective proteins involved in inflammatory processes, and it was able to avoid smooth muscle contraction impairment, preventing the LPS-dependent modification of some proteins involved in intestinal smooth muscle contraction [45]. Some of the most significant effects of inulin-type prebiotics are summarized in Table 1. 

Because of their antioxidant and anti-inflammatory activity, these substances could be considered in the prevention and treatment of GI disorders, in the pathogenesis of which oxidative stress plays a pivotal role. 

### 3.2. GOS

Less evidence is present regarding GOS mechanisms of action on the GI system. GOS are generally metabolized by numerous bacteria that possess β -galactosidase, which are able to digest them. For this reason, β-GOS, because of their specific bond, are more selective than plants’ GOS (α-GOS) for specific bacterial growth, particularly the Bifidobacterium species, as demonstrated in vitro [48,49]. 

To date, clinical studies are limited. An interesting cross-over control study involving 59 healthy volunteers compared two types of β-GOS (a novel mixture of GOS originating from a probiotic versus GOS produced by industrial β-galactosidase) at doses of 7 g/day demonstrating that both promote a significant bifidogenic effect [50]. This was confirmed by a double-blind, 10-week cross-over study, conducted on a population of 40 elderly human volunteers, in which a significant increase of fecal bifidobacteria was found, following supplementation with a lower dose (5.5 g/day β-GOS) [51]. 

Interestingly, it has been demonstrated that a β-GOS mixture plays a role in modulating immune function. In an interesting study, conducted on elderly subjects, β-GOS supplementation was found to increase the immuno-regulatory cytokine IL-10, with a significant reduction of IL-1β expression, compared to a placebo [51]. It has also been shown that this mixture of GOS is able to increase the blood level of interleukin 8 (IL-8) and C-reactive protein and to improve Natural Killer (NK) cell activity [51]. Finally, an in vivo study showed that supplementation with GOS in mice also improved lipid metabolism, without a positive effect on glycemic metabolism and with a significant enrichment of Alloprevotella, Bacteroides, and Parasutterella in the mouse microbiota [52]. 

### 3.3. Lactulose

Lactulose has been used in clinical practice since 1957, and it is considered as “bifidus factor”, because it is able to increase the Bifidobacteria count [53]. Lactulose seems not to have bifidogenic effects on humans at doses of 3 g/day × 2 weeks [54], but it shows this effect at doses of 10 g/day × 6 weeks [55] and 20 g/day × 4 weeks [56]. In the study of Bouhnik et al., a 10 g/day lactulose administration did not show the same effect, although according to the authors’ opinion, the time of the intervention was perhaps too short (only 8 days) [57]. Lactulose administration is patient- and dose-dependent; not all subjects have the same beneficial response to lactulose administration and the microbiota composition, before the beginning of the consumption could influence the bifidogenic effect of the lactulose [58]. A recent in vitro study showed the dose–response relationship in administering from 2 to 5 g/day of lactulose on a computer-controlled model of the human bowel. At a low dosage (2–3 g), they observed an increase in Bifidobacteria, but not in Lactobacilli, and a low production of SCFAs, while the administration of the maximal experimental dose (5 g/day) determined the correct balance among the microbial population (Bifidobacteria, Lactobacilli and *Anaerostipes*) and SCFAs production. By further increasing the quantities (10 g/day), the authors observed a significant reduction in the production of butyrate and an increase in acetate, probably due to the growth of the bifidobacterium population, which usually produces acetate from its metabolism [59]. 

Sakai et al. recently conducted an interesting study on 26 healthy women to test the prebiotic effect of lactulose (1 g/day, 2 g/day and 3 g/day for 2 weeks) on defecation frequency, Bristol scale and number of bifidobacteria (measured by PCR). They observed a significant increase of defecation frequency and the number of Bifidobacteria, following the three doses of lactulose, suggesting that even 1 g/day of lactulose may have a prebiotic effect [60]. A randomized double-blind placebo-controlled crossover study of 60 healthy women demonstrated that the administration of 2 g/day of lactulose for 2 weeks had a significant effect on the frequency of defecation and an improvement in fecal consistency, associated with an increase in the number and the percentage of bifidobacteria in the stool (measured by PCR and next-generation sequencing, respectively). Both results seem to disagree with the abovementioned result of Terada et al. [54]. Lactulose administration resulted in an increase in Bifidobacteria, without affecting other types of bacteria normally present in the faecal microbiota [56]. However, the beneficial effects of lactulose on the composition of microbiota appear to return to their original level already after 7 days [61] from the last lactulose intake. Therefore, it is recommended to continue to ingest lactulose to benefit from its positive effects [56].

### 3.4. New Molecules with Prebiotic Effects

As mentioned above, other substances such as xilooligosaccharides, soybean oligosaccharides, isomaltooligosaccharides, lactobionic acid, resistant starch and polyphenols seems to exert prebiotic properties [5,12,13].

The prebiotic and bifidogenic actions of Xilooligosaccharides (XOS) were observed in a randomized clinical trial (RCT), in which an administration of XOS for 2 and 4 weeks determined, in both cases, an increase in Bifidobacteria and butyrate fecal concentrations [62]. XOS determined the selective growth of *Bifidobacterium lactis* and *Bifidobacterium adolescentis* in pure cultures [63], but this result was not able to describe the prebiotic fermentation in a complex microbial environment, such as the intestine [7]. Thus, XOS were also tested in a simulated colon model to observe its effect in the gut. Researchers found that the XOS fermentation by Bifidobacterium determined an increased butyrate and acetate production. The bifidogenic effects of XOS on *B. lactis* seemed to be more efficient than those of FOS alone in a colon model, suggesting that the association between XOS and *B.lactis* could generate a successful symbiotic product [64]. A 6-week randomized controlled trial, conducted on 20 healthy subjects, demonstrated that those who consumed 150 g rice porridge with XOS supplementation (1.2 g XOS once daily) had increased fecal bacterial counts of Lactobacilli and Bifidobacterium and a decreased Clostridium, without altered total anaerobic bacterial counts, compared to the control group, who ate only rice porridge, without supplementation [65]. The increase in lactobacilli was in contrast with the above results obtained in a pure culture [63] and with another study, conducted on humans by Finegold et al., where the authors showed no changes in lactobacilli counts, stool pH and SCFA production after XOS administration at different doses (1.4 g and 2.8 g) [66].

Less studies are present regarding soybean oligosaccharides, isomaltooligosaccharides, lactobionic acid, resistant starch, for which there are only in vitro studies or in vivo studies conducted on animal models.

Soybean oligosaccharides (raffinose, stachyose, verbascose) reach the colon intact, where they are fermented from microbiota, especially Bifidobacteria [57,67]. An in vitro study evaluated the fermentation and the prebiotic potential of soybean Okara on the human fecal microbiota of healthy volunteers. The results showed that the soybean by-product, Okara, is able to promote bifidobacteria and lactobacilli growth, after 4 h of fermentation, with a greater inhibition of harmful bacteria, such as Clostridia and Bacteroides. Moreover, Okara’s cell wall is more difficult to digest, compared to FOS, thus suggesting a more prolonged prebiotic effect than that of other prebiotics [68]. An in vivo study on mice showed that an intra-gastric administration of raffinose oligosaccharides improved both beneficial microbes and immunological functions [69].

Isomaltooligosaccarides (IMOs) promote lactobacilli and Bifidobacterium growth both in vitro and in vivo [70,71,72]. An in vivo study showed the effects of IMO, Green Tea extract (GTE) and a combination of IMO and GTE on mice for 12 weeks, after consuming a high fat diet, which was associated with dysbiosis. This synergistic effect of IMO and GTE has been shown to have positive effects on visceral adipose tissue, on the production of pro-inflammatory cytokines and on lipid and glycemic control, and it has also been shown to improve insulin, glucagon and leptin levels. Furthermore, this association also acts positively on microbiota (Lactobacilli, Bifidobacterium, Akkermansia Mucinifila and Roseburia) and improves the Firmicutes / Bacteroidetes and Prevotella /Bacteroidetes ratios [73].

Lactose-derived prebiotics have a great potential for gastrointestinal health, especially in the case of diarrhea or constipation, and the prevention of inflammatory bowel diseases (IBD) and colon cancer. This beneficial effect on the gut is increased when lactose-derived prebiotics are associated with probiotics [74]. Lactitol seems to be more palatable and as effective as lactulose in the treatment of chronic constipation, with fewer side effects [75]. For example, 10 g/day of lactitol, administered to healthy subjects, determines an increase of Bifidobacterium and of propionic and butyric acid production, as well as a decrease of fecal ph. This dose could determine, in a small percentage of patients, mild side effects, such as flatus and borborygmi, suggesting an interesting dose of this substance to obtain its prebiotic effects, without important gastrointestinal symptoms [76]. Ballongue et al. observed that lactitol fermentation and utilization are slower than those of lactulose [56]. A similar effect on Bidobacterium and a lower pH is associated with Lactosucrose, another lactose-derived product [77].

An in vitro study evaluated the prebiotic and anti-inflammatory properties of lactobionic acid (LBA), a bionic acid naturally found in “Caspian Sea yogurt” [78]. Goderska et al. used different concentrations of LBA, observing a proportional bacterial growth, especially for Lactobacilli and Bifidobacterium [79]. LBA seems to be resistant to digestive enzymes, so it reaches the colon intact, where it is fermented by microbiota [80], probably interfering with lactose absorption, which may cause a binding competition [79,81]. On the other hand, LBA also has anti-inflammatory properties, and in a study on mice, it was demonstrated that its administration is associated with a decrease of obesity and a better control of metabolic parameters [82].

Resistant starch (RS) is naturally present in cereal grains and in all starch-containing foods. RS is divided into 4 classes [12,83] for its digestion resistance. That capacity is influenced by the granule morphology, amylose-amylopectin ratio and interaction with other food components [12]. An interesting study has demonstrated that RS has a bifidogenic effect, increasing the concentration of Bifidobacteria, Bacteroidetes, Akkermansia and Allobactum species [12]. Moreover, other studies in vitro and on mice demonstrated that resistant starch determined an increase of short-chain fatty acids [84,85,86]. There are few studies on humans, although in adults, high amylose maize starch (HAMS) administrations seem to be prebiotic [87]. 

Glucomannans are neutral polysaccharides present in some plants, such as the Amorphophallus family (i.e., Konjac or Oncophyllus), Orchid and eastern white pine. Glucomannan, extracted from konjac (KGM), has a higher DP (about 6000), and it is commonly used in the food industry as a food ingredient in Europe (E425). Its flour has numerous beneficial effects on human health, such as improving blood cholesterol and glycemia and reducing constipation. Moreover, konjac glucomannan seems to be able to stimulate the growth of beneficial microorganisms in the human gut. Al-Ghazzewi et al. observed in vitro that konjac hydrolysate stimulates Lactobacilli and Bifidobacterium growth more than the inulin in UHT milk [88]. Several studies confirmed these data, also showing a reduction of *Clostridium perfringens* and *Escherichia Coli* [89,90,91]. Harmayani et al. tested glucomannan effects for 14 days on 32 mice, which were divided into four groups: (1) porang glucomannan (extracted from Oncophyllus), (2) konjac glucomannan, (3) inulin, and (4) cellulose, as the control group. The results of this study have shown that porang glucomannan is more soluble than konjac glucomannan, and it is able to inhibit E. Coli growth and increase Lactobacilli and Bifidobacteria. Moreover, SCFAs production also increased in this group, and cecal pH value decreased [92]. A recent in vitro study evaluated the differences in microbial growth (especially Lactobacilli, Bifidobacterium, Clostridium and Eubacterium) of Konjac Glucomannan (KGM), low density Konjac oligo-glucomannan (LKOG), High-density konjac oligo-glucomannan (HKOG), Porang glucomannan (PGM) and inulin, as a positive control. These authors have observed that Bifidobacterium increased after 6 h of KMG, HKOG, PGM and inulin and after 24 h of LKOG, while lactobacilli significantly increased after 48 h for LKOG and after 6 h for HKOG, KGM and inulin, but not following PGM treatment. On the contrary, Bacteroides were reduced after 72 h for all substrates, while clostridia were reduced after 24 h for LKOG and inulin and after 12 h for KGM and PGM. As a final result, the authors underlined that LKOG was selectively fermented by beneficial bacteria, with a higher butyrate production [93]. 

Polyphenols are the secondary metabolites of plants and represent a substantial component of several plant-based aliments and beverages, such as different types of fruits and vegetables, as well as wine, coffee and tea. As the small intestine absorption of ingested polyphenols is very low, most polyphenol compounds reach the gut microbiota intact, producing a modification in its composition by exhibiting prebiotic activity and antimicrobial action against pathogenic agents [94,95]. Both Cranberry supplementation and Concord grape and California table grape extracts (rich in proanthocyanidin) increase the abundance of *Akkermansia*-enhancing mucus secretion [96,97,98]. There are interesting data regarding red wine, which is rich in a polyphenol, resveratrol. It has been demonstrated that an intake of 272 mL per day over a 30-d period is responsible for a significant modification of the gut microbiota composition in patients with Metabolic Syndrome (MetS), with a significant increase of Bifidobacterium, Lactobacillus, *F. prausnitzii* and *Roseburia sp* levels [99]. Therefore, resveratrol seems to exhibit anti-inflammatory properties through the inhibition of pro-inflammatory mediators, such as cyclooxygenase-2 (COX-2), IL-6, Tumor Necrosis Factor-α (TNF-α), Nuclear Factor kB (NFkB) and Vascular-Endothelial Growth Factor (VEGF) [100]. In particular, COX-2 activity is inhibited by several different phenolic compounds, probably by binding to the enzyme [47].

Moreover, the polyphenols present in red wine and green tea are able to reduce Helicobacter Pylori pathogenicity, inhibiting the urease activity and the growth of this bacterium and destroying the bacterial cell membrane integrity [101]. An interesting randomized controlled double-blind crossover intervention study tested the effect of cocoa flavanols on the intestinal microbiota composition in healthy individuals, demonstrating a significant increase of Bifidobacteria and Lactobacilli populations and a reduction of Clostridia counts. Interestingly, the results of this study also demonstrated a significant reduction of serum triacylglycerol and C-reactive protein, also assuming an anti-inflammatory effect [102]. A study evaluating the effect of green tea, after 10 days of administration, showed an increase in bifidobacteria [103], and this result was also confirmed by a previous study, in which the subjects received a product containing 70% tea polyphenols three times a day for 4 weeks. In the latter study, a significant reduction in clostridium was also observed [104]. Table 2 summarizes all the mechanisms of action of the prebiotics mentioned in this review.

## 4. Prebiotics and Gastrointestinal Disorders

The interaction between dietary intake, microbiota and gastro-intestinal disorders, i.e., irritable bowel syndrome (IBS) and inflammatory bowel diseases (IBDs), is emerging, in part due to the development of more scientific and standardized approaches to examining dietary intake, microbiota and disease outcomes. Moreover, it is well known that there is a huge spectrum of microbiota composition among people, and several gastrointestinal diseases are characterized by different degrees of dysbiosis [61].

IBS is a functional disorder, in which microbiota is thought to play a pivotal role. In particular, the relative lower numbers of Bifidobacteria demonstrated in diarrhea-predominant IBS has suggested the use of prebiotics in its management [114]. These results could explain, in part, why a low FODMAPs (Fermentable Oligosaccharides, Disaccharides, Monosaccharides and Poliols) diet, which excludes these fermentable substances, is reported to ameliorate GI symptoms in patients with IBS, suggesting the importance of finding the right equilibrium of short-chain carbohydrates for the management of these patients [115]. This diet could nevertheless be useful for ameliorating symptoms, as it has been demonstrated that it reduced luminal bifidobacteria [116,117], which are negatively associated with pain in both healthy controls and IBS patients [118,119].

In two studies, no improvement in IBS symptoms was reported after FOS supplementation at doses of 6 g/day for 4 weeks and 20 g/day for 6 weeks [120,121]. In contrast, an improvement in symptoms with FOS was reported in a more recent RCT, in which 105 patients diagnosed with minor Functional Bowel Disorders were randomized into two groups to receive either 5 g/day of short-chain FOS or 5 g of a placebo (sucrose and maltodextrins) over a 6-week period [122]. Despite these data, evidence of prebiotic effectiveness in adult IBS patients is still controversial, as reported in a recent systematic review and meta-analysis of randomized controlled trials, in which the prebiotic type and dose significantly influenced symptom improvement and exacerbation [123]. As a matter of fact, there is some evidence that higher doses may have a negative effect on symptoms [120,121,124,125,126].

Actually, there are few studies on the effects of GOS on functional gastro-intestinal disorders: an interesting single-center 12-week parallel crossover trial, which used a β-GOS in IBS patients (Rome II criteria), showed a dose-dependent stimulation of Bifidobacteria at 3.5 and 7.0 g/day. The symptoms, measured as a global assessment, were significantly improved, compared with a placebo, in both groups receiving the prebiotic at different doses, but the 3.5 g/day dose resulted in lower symptom scores for flatulence, bloating, and stool consistency [127]. In a double-blind, placebo-controlled, crossover study, in which β-GOS^®^ treatment (2.75 g/day) was tested in 83 subjects who presented with GI symptoms (abdominal pain, bloating and flatulence), this prebiotic was able to significantly reduce the scores of symptoms, after two weeks of assumption [128]. 

As already mentioned, β-GOS have anti-inflammatory effects [51]. Thus, it could explain the possible beneficial effects of these prebiotics on IBS patients, in which a microscopical inflammation of intestinal mucosa was found [125]. A recent study in vivo tested a blended prebiotic, which contains FOS, GOS, inulin and anthocyanidin (a class of polyphenols), on mice, and it showed an anti-inflammatory power on Caco-2 cells and in IBS symptoms by modulating microbiota. These results show that the synergistic action of more than one prebiotic could perhaps have greater effects on gastrointestinal symptoms [129]. A recent meta-analysis that evaluated the effects of FOS and GOS on IBS symptoms showed that both types of prebiotics did not have important effects on symptoms except for GOS that provided a significant effect on global IBS symptoms but not on abdominal pain [130] Moreover, psyllium is usually used in chronic constipation due to its capability to retain water in the small intestine, tinning the stool and increasing the defecation frequency [111]. Psyllium has little effects on healthy subjects’ microbiota, but it has a great beneficial effect in constipated individuals. At the baseline, constipated subjects had higher levels of *Desulfovibrio* ssp., which has been associated with a reduced intestinal motility in an in vivo model. Moreover, the SCFAs concentration was also different between healthy and constipated subjects. After psyllium administration, microbial species associated with the production of SCFAs, such as *Fecalibacterium* and *Phascolarctobacterium*, increased, while *Christensenella*, associated with hard stools, decreased [111]. 

IBD is a chronic, relapsing, multi-factorial disorder, causing inflammation of the gastro-intestinal tract and affecting both the colon and the small intestine, including Ulcerative colitis (UC) and Crohn’s Disease (CD) [131]. The pathogenesis of IBD has not been fully understood, but both genetic and environmental factors, including gut microbiota, seem to be involved [126]. 

Indeed, there is a growing interest in the hypothesis that the gut dysbiosis can be related to the immune alteration associated with IBD, and most of the literature regarding the use of prebiotics in GI disorders explore their efficacy in IBD patients. It has been demonstrated that commensal microbiota is able to protect mucosa from inflammation by decreasing intestinal permeability and increasing epithelial defense mechanisms [122,126,132]. Antibiotic-mediated microbial manipulation has some efficacy, particularly in active CD and pouchitis, but cannot be chosen in the maintenance of remission because of the lack of long-term efficacy and side effects [133,134,135]. A novel treatment approach is represented by prebiotics that selectively manipulate gastrointestinal microbiota. In fact, in patients with chronic pouchitis, treated with 24 g per day of inulin, a significant reduction in the number of Bacteriodetes was reported [136]. In another randomized study, involving 103 Crohn’s Disease patients, who received FOS 15 g/day, no clinical improvement was reported, but a reduction of the IL-6 of lamina propria dendritic cells (DC) and an increase of IL-10 DC staining were observed [137]. In a single-arm study intervention, fructan administration determined an improvement of disease symptoms in 10 CD patients, correlated with an increase of the Bifidobacteria concentration and of the percentage of interleukin-10, positive dendritic cells those that express toll-like receptor-2 and toll-like receptor-4. This condition highlights a beneficial modification of mucosal dendritic cell function [138]. On CD patients, another study was also conducted, in which fructans administration showed a reduction in dyspeptic symptoms and in the levels of calprotectin, a bowel inflammatory biomarker, 7 days after the beginning of the intervention [139]. Joossens et al. conducted two studies to evaluate the effects of fructans supplementation: in the first one, the authors administered 20 g of fructans for 4 weeks to 17 healthy subjects, and they observed an increment of *Bifidobacterium longum* and *B. adolescentis* [140]. The second study of the same research group evaluated a 10 g administration of fructans to 67 subjects affected by mild IBD for 4 weeks, and they had no effects on *F. prausnitzii* and *B. adolecentis*, while *Rhamnococcus gnavus* and *B. longum* increased, with a significant improvement of disease symptoms [107]. Furthermore, two studies included in a recent metanalysis—one focused on prebiotics in an adult population and the other in children and young individuals—reported that the response to mesalazine is positively influenced by prebiotics through a mitigation of intestinal inflammation [141]. 

Hafer et al. conducted a pilot study on 31 subjects suffering from IBD (both UC and CD) and found that a 10 g lactulose administration did not show any beneficial effect, except for an improvement of the clinical symptoms and the quality of life of patients with CU, without significant modifications at the endoscopic level [142]. As Fellerman et al. reported, this discrepancy could be related to a defensin deficiency in IBD, which is reversible only in UC and not in CD patients, following lactulose administration [143,144].

Arabinooligosaccharides (AOS) seem to reduce inflammatory conditions in UC subjects, even if there are only preliminary results. Interestingly, an in vitro study showed that AOS, as well as FOS, are able to stimulate an increase of Lactobacilli and Bifidobacterium in fecal microbiota derived from UC patients. The FOS effects were clearly positive in increasing the content of both Bifidobacterium and Lactobacilli, while for AOS there was a positive trend, but the evidence was not so strong. However, AOS determined a significant reduction, especially in *Firmicutes*, but also in *Bacteroidetes and Desulfovibrio* [108]. The increase of Lactobacilli and Bifidobacterium in the UC patients’ fecal microbiota was associated with a higher production of acetate, which determines a decrease in pH, probably contributing to the amelioration of inflammation and prevention of flare-ups [108]. 

It has been demonstrated, in a 4–month RCT, that psyllium husk has beneficial effects in patients with inactive UC, improving symptoms, such as bloating, diarrhea, abdominal pain, urgency, incomplete evacuation and constipation, compared to the baseline [145]. A one-year RCT tested the prebiotic effect versus the drug effect in UC patients, divided into a psyllium treatment group, a mesalazine group and a psyllium and mesalazine group. They observed that the synergic effect determined better but not significant results, compared to the other two groups. However, the psyllium group increased the fecal levels of butyrate [112]. Moreover, starch seems to have other gastrointestinal beneficial effects, such as the increase of the stool bulk, promoting regular intestinal movement [146], a decrease of the cecal pH [146] and a prevention of the mucous layer degradation in the colon [147].

Among gastro-intestinal disorders, colorectal cancer would also seem to be a therapeutic target of prebiotics [131]. A systematic review analyzed some clinical trials to underline the effect of some prebiotics (fructans, lactulose, and resistant starch) on colorectal cancer biomarkers, but they did not find any positive association between prebiotic consumption and a reduction of colorectal cancer biomarkers, except for lactulose administration—researchers found that it decreased the adenoma recurrence [148]. On the other hand, the potential effects of the use of a symbiotic therapy (*Lactobacillus Rrhamnosus* and *Bifidobacterium Lactis* plus inulin) would seem to improve the integrity and the function of the epithelial barrier, as well as reduce the rate of cell proliferation in patients with colon cancer [149,150]. However, as our knowledge of gut microbiota improves, it seems that other microorganisms could benefit from prebiotic administration, such as *Clostridium coccoides* or the *Eubacterium rectale* cluster, which includes bacteria-producing butyric acid, a beneficial metabolite for gut functionality that is potentially protective against bowel cancer [10,23]. Interestingly, some degradation products of prebiotics are able to promote beneficial effects [151,152] and to have protective effects on colonic epithelial cells during the progression of colorectal cancer, inhibiting the nuclear factor kappa B activation and the histone deacetylation [153]. Butyrate seems to have a protective effect in the prevention of colonic cancer cell proliferation by provoking apoptosis through the induction of autophagy and by blocking the endoplasmic reticulum stress response [154]. 

Dietary polyphenols have also been studied in relation to colon cancer prevention, even if the data are conflicting [155]. In animal models, it was demonstrated that resveratrol supplementation reduced bacterial enzyme activity, such as the activity of b-glucuronidase, b-glucosidase, b-galactosidase, mucinase and nitroreductase, and this decrease was linked with a major decline in colonic tumor occurrence [156]. 

## 5. Side Effects

To date, no serious side effects have been reported after the consumption of most prebiotics.

Oligosaccharides and polysaccharides, not hydrolyzed by intestinal enzymes, are fermented by intestinal microbiota. Therefore, the only side effects of prebiotics, mainly related to their osmotic effect, are diarrhea, bloating, cramps and flatulence. The length of prebiotic chains plays a pivotal role in influencing the development of side effects. The ingestion of inulin, in a liquid or solid meal, usually does not have serious side-effects [33,157]. However, generally, prebiotics with a shorter chain are more likely to have side effects probably because the shorter inulin molecules are metabolized mainly in the proximal colon and undergo faster fermentation. Additionally, the prebiotic dose can influence its safety profile and, in fact, low doses (2.5–10 g/day) can cause flatulence while high doses (40–50 g/day) are responsible of osmotic diarrhea [148]. 

In cases of diarrhea—predominant IBS prebiotics are not recommended due to their stimulation of gas production in the colon, determining an increasing of bloating in these patients [158]. Additionally, in constipated patients, there is not evidence to support the use of prebiotics [5]. For IBD patients, instead, prebiotics like inulin have a benefic role in reducing the gut mucosal inflammation so they can be a useful tool for this pathological condition [159]. At high doses (10–20 g/day), lactulose could induce inconvenient symptoms such as flatulence or bloating as well as laxative effects for its osmotic properties [160]. The most significant adverse effect of lactulose is flatulence, which is not present after the administration of 2 g/day [60] while it appears in a dose-dependent manner increasing the dose from 3 g/day to 5 g/day [161]. In another study a single-dose lactulose challenge was administered to observe the effect of this substance on microbial stability; the authors tried to increase *E. Coli* concentration in order to reduce enteropathogens (such as *Salmonella thypimurium*) using H2 produced by lactulose fermentation in the colon. They did not observe this effect while the single dose of 50 g caused significant side effects such as diarrhea, bloating, borborygmi and abdominal pain [162]. Moreover, in this study, they did not observe the bifidogenic effect of lactulose because of the single dose used; these data confirmed that the prebiotic effect of Lactulose is dependent on a prolonged administration, as suggested in the previous study of Bouhnik et al. [55]. In the same way, also the fermentation of lactose-derived prebiotics produce gases responsible for bloating, flatulence and abdominal pain in patients [81].

Finally, XOS, were well tolerated for 8 weeks intervention without side gastrointestinal effects at both doses of 1.4 and 2.8 g/daily [66].

## 6. Conclusions

Dietary intake of prebiotics seems to positively affect the intestinal microbiota not only promoting the growth of beneficial intestinal bacteria, but also producing metabolites that are potentially protective of gut functionality. Fructans (especially FOS and inulin) and galactans (GOS) are the most studied prebiotics in the literature while further investigations are needed to better understand the possible benefits of other prebiotics, such as Lactulose, Xylo-Oligosaccharides, Arabino–Oligosaccharides, Resistant Starch or soybean Oligoschaccharides. Interestingly, there is recent evidence of polyphenols, which could represent a novel target in the management of several pathological conditions, being involved in GI disorders. 

Moreover, because of their antioxidant and anti-inflammatory activity, prebiotics, especially Inulin, could be used in the prevention and treatment of GI disorders in which inflammation and oxidative stress play a pivotal role in their pathogenesis. Finally, preclinical and clinical studies have shown that various dietary prebiotics exert several benefits on distinct diseases connected with intestinal microbiota modulation, although further experimental evidences are still necessary to better understand the molecular mechanisms involved and the clinical efficacy. In fact, the few meta-analyses concerning the use of prebiotics in GI diseases (Table 3) underline the scarcity of data supporting an evidence-based use of these substances which shows controversial effects in alleviating GI symptoms.

## Figures and Tables

**Table 1:** In vitro effects of inulin-type prebiotics.

Prebiotic	Effect	Reference
Inulin-type fructans	Stimulate the activity of the glutathione S-transferases of antioxidant enzymes	[29]
Inulin	Induces scavenger activity in the radical O_2_	[30]
Inulin	Prevents lipid peroxidation in the stomach	[46]
Inulin and other “sugar-like” elements	Replace vitamin C as a dietary supplement and/or limit its degradation	[47]
Inulin-type prebiotics	Inhibit the degradation of ascorbate	[47]
Inulin	Causes a protective effect on LPS-induced damage of colonic muscle	[41,42]

LPS, Lipopolisaccharide.

**Table 2:** Mechanisms of action of prebiotics.

Type of Prebiotics	Structure	Mechanisms of Action
**Fructans (Inulin and Fructooligosaccharides, FOS)**	Fructosyl-fructose β (2X1) glycosidic bonds (FOS DP 2–9; inulin DP 2–60) [25].	↑ Lactobacilli and Bifidobacterium (especially *B. longum subsp. Longum, B. pseudocatenulatum, B. bifidum and B. adolescentis*) growth [22,26]. ↑ SCFAs production [26]. Act indirectly as a scavenger of Reactive Oxygen Species (ROS), thanks to the action of SCFAs and can stimulate the activity of the glutathione S-transferases (GSTs) of antioxidant enzymes [42]. Contrast the oxidative stress induced by LPS in human colonic mucosa [42].
**Galactooligosaccharides (GOS)**	Commercially produced by the enzymatic activity of β-galactosidase transferase on lactose (DP 2–8). It is a galactose polymer with a terminal β-linked glucose monomer [105].	↑ Bifidobacterium [50] and fecal Bifidobacteria concentration growth [51]. Can modulate immune function: ↑ Cytokine IL-10, interleukin 8 (IL-8) and C-reactive protein, improve Natural Killer (NK) cell activity, and ↓ IL-1β expression [51]. Improve lipid metabolism [52]. Enrich the mouse microbiota of *Alloprevotella, Bacteroides, and Parasutterella* [52].
**Lactulose**	Synthetic disaccharide Galactose–fructose β (1–4)-linked [55].	At a low dosage (2–3 g/day), ↑ Bifidobacterium count [53,59], but not Lactobacilli, and determines a low production of SCFAs; 5 g/day determines the correct balance among the microbial population (Bifidobacteria, Lactobacilli and Anaerostipes) and SCFAs production, while 10 g/day ↓ butyrate production and ↑ acetate [59].
**Lactobionic acid**	A gluconic acid bonded to a galactose [78].	↑ Lactobacilli and Bifidobacterium growth [79]. Has anti-inflammatory properties, ↓ obesity and improves metabolic parameters [82].
**Xilooligosaccharides (XOS)**	Xylose units linked by β (1–4) bonds, with a DP of 2 to 10 [106].	↑ Bifidobacteria (especially *Bifidobacterium lactis* and *Bifidobacterium adolescentis* [63]), Lactobacilli [65] and butyrate fecal concentrations [62]. ↓ Clostridium growth [65]. No changes in lactobacilli counts, stool pH and SCFAs production [66].
**Arabinooligosaccharides (AOS)**	α (1–6)-linked backbone of L. Arabinosyl residues, which can be single- or double-substituted with α (1–2)- and/or α (1–3)-linked L-arabinosyl residues [107]	↑ Lactobacilli and Bifidobacterium growth [108]. ↓ *Firmicutes, Bacteroidetes and Desulfovibrio* [108]. ↑ Production of acetate that determines a decrease in pH, probably contributing to the amelioration of inflammation and prevention of flare-ups in UC patients [108].
**Soybean oligosaccharides**	Tri, tetra or pentasaccharide galactose–sucrose α (1–6)-linked [67]	↑ Lactobacilli and Bifidobacterium growth [68]. ↓ *Clostridia and Bacteroidetes* [68]. ↑ Immunological functions [69].
**Isomaltooligosaccharides (IMO)**	Gluco-oligosaccharides, with an α (1–6) bond and DP between 2 and 10 (di-, tri- and tetrasaccharides) [71,109,110].	↑ Lactobacilli and Bifidobacterium [70,71,72,73], *Akkermansia, and Roseburia* [73] growth. Improve *Firmicutes / Bacteroidetes* and *Prevotella / Bacteroidetes* ratios [73]. Show positive effects on visceral adipose tissue, on the production of pro-inflammatory cytokines and on lipid and glycemic control, improving insulin, glucagon and leptin levels [73].
**Resistant starch**	Glucose polysaccharides consisting of amylose (α (1–4) bonds) and amylopectin (α (1–6) bonds) [12].	↑ Bifidobacteria, Bacteroidetes, *Akkermansia and Allobactum* species [12]. ↑ SCFAs production [84,85,86].
**Glucomannan**	Mannose and glucose at a molar ratio of 1.6:1, with little residues of galactose or acetyl groups [88].	↑ Lactobacilli and Bifidobacterium growth [88]. ↓ *Clostridium perfringens* and *Escherichia Coli* growth [89,90,91]. ↑ SCFAs production [92]. ↓ Cecal pH value [92]. Improves blood cholesterol, glycemia and reduces constipation [88].
**Psyllium**	Highly branched and gel-forming arabinoxylan, a polymer rich in arabinose and xylose [111].	↑ *Fecalibacterium and Phascolarctobacterium* growth, associated with SCFAs production [111]. ↓ *Christensenella*, associated with hard stools [111]. ↑ Butyrate fecal concentration [112].
**Polyphenols**	Hydroxylated aromatic rings or phenol rings [113].	↑ Lactobacilli and Bifidobacterium [99,102,103,104] *Akkermansia* [96,97,98], *Roseburia* and *F. Prausnitzii* [99] growth. ↓ *Clostridium* growth [102,104]. Offset *Helicobacter Pylori*-inhibiting urease [101]. Inhibit pro-inflammatory mediators: cyclooxygenase-2 (COX2), IL-6, Tumor Necrosis Factor-α (TNF-α), Nuclear Factor kB (NFkB) and Vascular-Endothelial Growth Factor (VEGF) [47,100]. Reduce serum triacylglycerol and C- reactive protein [102].

↑: increase; ↓: decrease.

**Table 3:** Summary of most relevant meta-analysis results of prebiotics’ effects in irritable bowel syndrome (IBS), inflammatory bowel diseases (IBD) and colon cancer.

	Meta-Analysis	Eligible RCTs	Prebiotics Analyzed	Effects
**IBS**	Wilson et al. 2019 [123]	11	non-inulin-type fructan prebiotics	Improvement of flatulence severity score
			inulin-type fructans	No benefits
	Ford et al. 2018 [158]	2	Fructooligosaccharides	No results on symptoms
		1	GOS	Reduction in mean global symptoms but not in abdominal pain
	Asha MZ et Al. 2020 [163]	3	partially-hydrolysed guar gum and fructooligosac	no benefits
**IBD**	Astó et al. 2019 [140]	2	Inulin—type fructans	Prebiotics help mesalazine to mitigate intestinal inflammation
**Colon cancer**	van Dijk M. 2016 [164]	4	prebiotic fibers	insufficient data
